# Three Memoirs on the Development and Structure of the Teeth and Epithelium

**Published:** 1841-10-01

**Authors:** 


1. THREE MEMOIRS ON THE DEVELOPMENT AND STRUCTURE OF THE TEETH AND EPITHELIUM, Read at the Ninth Annual Meeting of the British Association for the Encouragement of Science, held at Birmingham, in August, 1839; with Diagrams exhibited in illustration of them. By *Alexander Nasmyth*, F.L.S., F.G.S.. Member of the Royal College of Surgeons, &c. 8vo. pp. 47. London: J. Churchill, 1841.

II. ODONTOGRAPHY; OR A TREATISE ON THE COMPARATIVE ANATOMY OF THE TEETH; THEIR PHYSIOLOGICAL RELATIONS, MODE OF DEVELOPMENT, AND MICROSCOPIC STRUCTURE IN THE VERTEBRATE ANIMALS. Illustrated by upwards of 150 Plates. By *Richard Owen*, F.R.S. &c. Parts I. and II. H. Bailliere, London, 1840.

THE feelings that have arisen between the gentlemen whose works lie before us, and the controversy that has engaged the attention of the public are calculated to pain every lover of science. Physiologists would seem to be a genus irritabile as well as poets, and the more minute and microscopic the point, the more violent the dispute about it. It is neither our habit nor our taste to enter into personal quarrels, and we shall not, in this instance, infringe our custom. We trust that reflection will convince Mr. Owen and Mr. Nasmyth that they consult their dignity by avoiding personalities, and that both suffer by discussions which only amuse the idle and malicious.

Without indulging in any further observations, or meddling with a controversy, unpleasant at the best, we shall content ourselves with presenting, in the first instance, a pretty complete view of Mr. Nasmyth's researches.

Mr. N. observes at starting, that the application of the microscope to the study of the fossil teeth has opened a new field of inquiry. Mr. N. continues,—" In endeavouring to form a classification of the animal kingdom, having for its basis the peculiar disposition and arrangement of the internal organization of the teeth, I was led to the discovery of a structure, which has hitherto not been noticed. Purkinje and Fränkel state, that ' the proper dental substance consists of a uniform structureless substance, and of fibres passing through it.' The writings on the subject by Retzius, Müller, and others, leave us to conclude that the interfibrous substance does not present any traces of peculiar conformation:—but I am disposed to believe that it is not only organized, but so differently and characteristically so in different animals, as to be capable of affording valuable aid to the naturalist in classifying the animal kingdom." Mr. Nasmyth enjoins those who would pursue these investigations to make use of a magnifying power of one tenth of an inch focal distance, and of the most perfect kind, with an acromatic condenser of the light.

Not only has Mr. Nasmyth satisfied himself of the cellular character of the interfibrous structures of the teeth, but he has also made researches into the structure and composition of the fibres of different teeth, and has generally found that these present an interrupted or baccated appearance, as if they were made up of different compartments,—an obvious concomitant of the cellular structure of the interfibrous material. The size and relative position of these portions or divisions of a fibre differ in various series of animals. In the human subject, for instance, each compartment of the fibre is of an oval shape, and its long small extremity is in apposition to the one next adjoining. The long axis of the oval corresponds with the course of the fibre. In some species of the monkey tribe the fibre appears to be composed of two rows of compartments parallel to each other. In the orang utan the fibre is composed of rhomboidal divisions, and in the baboon they are oval like those of the human subject, and the surfaces of the long axes are in apposition. In fact, each class of animals seems to have a distinct characteristic appearance, but all are similar in respect to the general baccated appearance.

In the tusk of the mammoth, the structure seems laminated, and there are other circumstances, which tend to bear out the opinion that teeth ordinarily are so. Mr. Nasmyth observes :—

" If we suppose that ivory consists only of fibres surrounded by a structureless material, passing from the centre to the circumference, we must conclude that at right angles to their course it would be most difficult of fracture; but the contrary is the fact, as is exemplified by the natural separation of the lamellæ of the tusk of the mammoth in a direction contrary to the course of these fibres, as well as by the mode adopted by ivory cutters of making sections corresponding to the vertical, and not to the transverse diameter of the tooth. Many other facts might be here adduced, as well as phenomena accompanying the decay of these organs, were this necessary or relevant to the business of this section. All systems of dental structure which have hitherto been propounded have failed, I think, to explain facts of daily occurrence, but they may be accounted for, I

venture to assert, by the cellular organization of the interfibrous substance which has been improperly termed structureless, and by the peculiar baccated arrangement of the fibres." 8.

*The Enamel.*—"According to the views of Retzius, Purkinje, and the recent investigators of the structure of the teeth by the aid of the microscope, the enamel consists of fibres running in a direction from the centre to the circumference of the tooth. Both Retzius and Purkinje have given delineations of the course of these fibres. On making a section of the enamel, in a direction parallel to the transverse diameter of the tooth, the appearance as described by these writers is observed, and they are said to be seen to terminate in an hexagonal form beneath the investing crusta petrosa. If, however, a different section of the enamel of the human tooth be made; for instance, one near the surface, parallel to the vertical direction or long axis of the tooth, an appearance presents itself which has induced me to take a different view of the nature of the structure of the enamel. The appearance to which I now allude is represented at [Plate C. 9,] figure marked Structure of the enamel, No. 1. It will there be found that the section of the enamel presents compartments or divisions, but of a different character from those I have already spoken of as existing in the interfibrous substance of the ivory. Each compartment of the enamel is of a semicircular form, and the convexity of the semicircle or arch looks upwards towards the free external portion of the tooth. The vertical section, [Plate C. 9,] enamel No. 2, gives the appearance of these cells as seen in that direction.

In the sections both of enamel and ivory, there will be always observed near the margins isolated cells, which admit of their form and appearance being carefully studied." 9.

*The Pulp.*—Mr. Nasmyth observes that the pulp is cellular throughout its entire structure. The surface of it, that portion where it's transition into ivory takes place, presents a beautiful reticulated cellular appearance, and this conformation is believed by Mr. N. to be essentially concerned in the development of the ivory.

An analogous reticular appearance is displayed by the internal or productive surface of the capsule.

The membranous investment which covers the enamel of the teeth of man and other simple teeth, displays a similar arrangement.

"Thus," says Mr. N., "there is a remarkable uniformity in the structure of the formative tissues of the tooth, and of the dental substance itself; for not only is the interfibrous material cellular, but the surface of the pulp, which is the organ for the production of the ivory, and the internal or productive surface of the capsule, also uniformly present a reticulated or cellular appearance.

My researches have, I venture to hope, also established a new and beautiful instance of the harmony of the laws of nature in demonstrating the fact of the uniformity of the products of the capsule: for not only is the enamel uniformly provided with an external covering, but there is also a membranous investment of the crusta petrosa itself. I think, also, that we must be compelled to allow the uniform presence of a fourth tooth-bone substance, the existence of which is more constant in all animals, either normally or anormally, than any of the other three hitherto recognized textures." 12.

Such is the substance of the first of the memoirs before us. The second is intended to lay before the public, through the medium of the Association, Mr. Nasmyth's conclusions in reference to the following subjects:—1st, on the covering of the enamel; 2ndly, on the structure of teeth; 3rdly, on the structure of the pulp and its relation to the development of the ivory.

Mr. Nasmyth first dwells on the fact that the enamel is, in all instances, covered with crusta petrosa. He adds:—"On the incisor of the calf and several other simple teeth, I have also distinctly traced in this layer of crusta petrosa, superimposed on the enamel, the corpuscules of Purkinje, analogous to those found in bone. I possess preparations of teeth of the human subject, and simple teeth of the herbivora and carnivora, showing this structure in a clear and unequivocal form."

Reverting to the fibres of the ivory, and the "baccated" appearance they present, he observes:—"To indicate the true theory of the formation of ivory, nothing more is required than the display of these appearances. No 'excreted' or 'exuded' substance can possibly present an animal tissue arranged in regular connected cells. It is quite evident that these cells, whilst receiving a supply of earthy matter during the process of transition must remain in connection with, and indeed continue to form part of, the pulp. It would be absurd to suppose that a regularly cellular structure can be 'excreted;' but it would be still more ludicrous to maintain, that after such cells have been excreted, that is to say, after all connexion between them and the pulp has ceased, they still possess the power or means of deriving from the blood the materials requisite for their transition into ivory, and of carrying on that process in their isolated state."

"The general appearance of the fibres thus treated is exactly similar to that of the fibres of cellular tissue generally, and the diameter of each corresponds exactly to the diameter of the calibre of the tube, which, according to Retzius, is pervious, although at the same time he says that it is always more or less filled with earthy matter. In fact, the tubes have been said to be principally visible by means of their contents, the reason of which appears to me obviously to be, that these contents are the only part of them which actually exist.

In order to separate the animal matter from the osseous substance of the tooth, I submitted thin slices of many different kinds of dental bone to the action of a solution of caustic potash, for a period sufficient to dissolve and remove the organic tissue; but the brittle nature of the residue, the difficulty of washing it without breaking down its structure, and the great opacity of the sections which had been thus treated, deprived this experiment of any striking results illustrative of the internal organization of teeth; but the appearances presented in its progress were all such as to favour the conclusion that the structure of the ivory is essentially cellular.

Having convinced myself of the existence of the peculiar cellular structure of the tooth, I entered with great interest on an examination of the organ by which it is produced, viz. the pulp.

On examining the internal structure of the pulp generally, the number of minute cells presenting themselves in a vesicular form is very remarkable; they seem to constitute indeed the principal portion of its bulk, These vesicles vary in size from the smallest perceptible microscopic appearance, probably the ten-thousandth part of an inch in diameter, to one-eighth of an inch, and are evidently disposed in different layers throughout the body of the pulp. They are of various shapes." 19.

When thin layers of macerated pulp are examined, they present an irreglar reticular appearance, and are found to be interspersed with granules. The parenchyma is traversed by vessels of which the direction is generally vertical.

Mr. Nasmyth conjectures that the cells of the pulp are filled either with air or fluid, he cannot distinguish which. He examines the question if the ivory be simply a product of the pulp, or a transformation of its substance. He says,—although it is distinctly demonstrated that the interfibrous substance of the ivory is formed by the deposition of osseous matter in the cells of the reticular surface of the pulp, I cannot boast of being able fully to unveil that interesting process. The cells for the reception of the earthy matter are displayed, but—how is this matter arranged in those cells? How is it, in the first place, derived from the blood and introduced into them? What are the causes of the characteristic varieties of the interfibrous cellular substance in different classes of animals? What is the precise degree of importance of the reticular cellular organization observed on the surface of the pulp with regard to the process of transition, besides the fact that it presents cells into which the earth is deposited? Do these reticular cells form a system containing circulating fluids, from which the osseous material of the tooth is eliminated? How are these cells connected together, both in their transition state and in the pulp, as well as when they have passed into the state of ivory? These, and many other similar questions, remain to be solved before our comprehension of the process of the formation of ivory can be said to be complete.

He proceeds to develop his own ideas on the formation of the ivory. On the surface of the pulp are found innumerable detached cells, with central points. Generally, these cells form a regular and complete coating, studded with points, which are placed at intervals, corresponding in extent to those between the fibres.

These points are rendered visible from the greater opacity of the intermediate material, and will be seen to reflect or absorb the light, according to the difference in the focal distance. A comparison between the superincumbent perfect ivory, and the formative surface of the pulp beneath, is always easy, because portions of the former, at an early stage at any rate, remain adherent to the latter, and fragments of the dental bone are found strewn over it, more especially in human teeth. The cellular conformation of these fragments is always evident, and in size and appearance they are perfectly accordant with the cells of the pulp. At an early stage of dental development, the reticulated or cellular appearance of the pulp is particularly beautiful. When merely a thin layer of ossific matter has been deposited on its surface, it may with great facility be drawn out entire, together with the former, laid on a glass, compressed a little, and then examined with the high powers of the microscope. The different layers of cells will be seen, and the transition into ivory may be observed.

"It appears to me," observes our author, "that the framework of the reticulation, or cells of the pulp, constitutes the fibres of the tooth, which, while in this state, are spirally coiled, and fit into one another. At all events, the diameter of these fibres of the reticulations is precisely that of the fibres of the ivory; the points or projections rising from the framework correspond to the centres of the cells, and may be traced to belong to their structure. The fibres composed of granules of animal matter, and which I describe as the framework of the reticulations, become, upon the deposition of ossific matter within the cellules of those reticulations, the fibres of the ivory. The only change which they appear to undergo during the process of transition, is, that they are then drawn out from the coiled-up state in which they exist between the collapsed cells of the reticulations into the more longitudinal but still spiral form in which they are found in the ivory." 29.

He is inclined to believe that this convoluted fibre is made up of single successive granules, which are developed one after the other from the body of the pulp, until the fibre is complete. The manner in which the osseous matter is deposited in the cells of the interfibrous substance, Mr. N. has not been able to discover. It would appear, however, that these cells are subdivided into minute cellules, for they present the appearance of being filled with granules in certain progressive stages of development. In whatever aspect we view the formative organs of the tooth, and the dental tissues themselves, and whether we examine the latter during the process of their development, or after their formation has been completed, we are everywhere met by appearances which denote a cellular or reticular arrangement.

Mr. Nasmyth adds :—

"I think that the view I have taken of the subject more satisfactorily explains than any other, facts of daily experience. The laminated arrangement of the osseous cells explains the concentric fracture of the tusks of the mammoth, and of other teeth, when left to decompose spontaneously. The cells being in imbricated apposition, and held together by earthy salts, being moreover arranged in layers conformably with the periphery of the pulp, must be regarded as concentrically laminated. The existence of this structure explains the phenomena daily noticed by ivory-cutters, and also Mr. Hunter's experiments of feeding animals with madder, the result of which is incompatible with any other theory of the structure of dental bone.

No view hitherto taken of the structure of dental bone has afforded a satisfactory explanation of the ordinary morbid appearances of the tooth, but many of these I think may be explained, if we regard the latter as cellular. Still I do not conceive that I have in any way exhausted the subject; far from it; I am quite alive to the imperfect nature of my researches, and am prepared for correction on many points, when more extended and varied investigations shall have been undertaken.

I find that Schwann, in a recent work, teaches that all the primary tissues of the animal frame are cellular, and has given to the world some remarkably interesting details on this subject. He says that he has remarked the characteristic 'cellular nuclei,' or elementary cells, on the enamel-membrane, that they are continued in minute fibres, and that these are similar to the epithelium cylinders in mucous membranes. He notices what he calls cylindrical cells on the surface of the pulp, and he supposes that these cylindrical cells of the pulp are the fibres of the tooth in their first stage, which does not at all coincide with my observations. He regards the dental substance simply as the ossified pulp, whilst my observations lead me to conclude that the cells of the ivory are altogether a distinct formation. He acknowledges, however, that he is ignorant of the process of transition, and he regards the dental pulp as a simple cartilage. In fact, he starts with a ready-made hypothesis, and founds his opinion rather on the observations of others, and on the inferences he draws from them, than on his own actual researches: with respect to what he himself gives as his own, it accords for the most part with the details I have just communicated." 33.

An account of Schwann's researches follows, but we need not insert it.

The *Third Memoir* contains a notice of Mr. Nasmyth's researches into the Structure of Epithelium. After alluding to the opinion first broached by Leeuwenhoek, that epithelium consists of scales, which are found on the surface of all mucous and serous membranes, as on the inner membrane of the vascular system, Mr. N. proceeds to describe the—

*Structure of Epithelium generally.*

The epithelium is a layer of substance destitute of vessels, which covers the vascular surface of mucous membranes. If the surface of a mucous membrane (for instance, the conjunctiva or the buccal) of a living animal be slightly rubbed, it will be found, on microscopic examination, that numerous small particles have been detached from it. At the first glance they present precisely the appearance of scales, for they are flat bodies with a thick portion or nucleus in their centre, and with very thin and transparent margins.

They are found not unfrequently with a curved margin and without a central spot or nucleus, and their surface often presents numerous transparent points, with very fine lines. The nucleus of the scale generally contains a small body which has been termed the nucleus-corpuscle. But by this simple method of observation we do not obtain an insight into their true structure. If we remove the secretion from the surface of an irritated mucous membrane, we shall find another class of bodies which differ from these first mentioned, in being smaller and more globular. They have a nucleus of the same size as those of the so-called scales, and also a nucleus-corpuscle, but the surrounding structure is in the form of a cell, and is much smaller. Here and there may also be observed a nucleus with its accompanying corpuscle, lying in substance which presents no appearance of a cell. The structures here described may also be seen on a careful examination of a section of the epithelium and mucous membrane of a young subject. On the surface of, and in immediate apposition to, the mucous membrane are seen numerous nuclei, which more externally are surrounded by a cell; and on approaching still nearer to the surface, we find this cell, from having increased considerably in size, and become compressed, assuming the appearance of a scale, which retains the nucleus, and its corpuscle of primitive size. The various stages of the development of the epithelium may be satisfactorily traced, by removing, after a short maceration, the layer of epithelium from the under surface of the tongue of a young calf, and placing it upon a piece of glass, when, if the external surface of the object be brought into focal distance, large scales only with their central nuclei are observable; but if the object be approximated to the glass, so as to bring the internal part of it into the proper focal distance, numerous small scales are brought into view; and if the object be still more approximated to the lens, so as to bring its internal surface into the proper focal distance, numerous rounded cells become apparent.

In the fœtus, the defined and well-formed scales of the epidermis, are not unfrequently distinctly seen externally; the *rete Malpighii* consists of newly-formed cells, and between the two may be observed other cells in a state of progressive development. On the surface of the vascular mucous membrane minute cells are found with a nucleus in their interior, round which the cells grow; and this, in short, is the process of development of the minute bodies which constitute the epithelium.

The component parts of the epithelium are connected, according to Mr. Nasmyth, in this manner. The cells on the surface of the mucous membrane are separated from each other by considerable spaces, which are occupied by a gelatinous substance, interspersed with minute granular bodies. But the scales forming superficial layers of the epithelium are separated by very minute linear spaces, but are still connected together by a translucent, gelatinous substance.

This latter displays considerable elasticity, as is easily rendered evident by an attempt to lacerate the epithelium in a moist state, if the latter be examined at the same time by the aid of a magnifying power. Each time that the laceration is attempted, the membrane yields, and the scales separate to a certain extent; but regain their original position, on the cessation of the effort to draw them apart. In some instances a fibrous structure is evident in the gelatinous substance between the scales.

The scales towards the free surface are distinctly observed to overlap. The gelatinous substance above alluded to, presents distinct granular bodies, which give to the epithelium, *en masse*, a rather dense aspect, the individual scales being sometimes covered by these granules; the latter can, however, be separated from the scales by compression; by which means, indeed, the granules themselves may be made entirely to disappear. In certain parts of the epithelium of the calf, distinct fibres are observed, which pass over the surface of the scales, and connect them together, thus forming a very delicate net-work. This appearance is most evident upon compression of the thick epithelium on the anterior part of the alveolar arch of the upper jaw.

In these cases, where the small scales, or small clusters of scales, are being continually thrown off, as on the surface of the body, and of the mucous membranes of man and animals generally, the scales composing the external layer will be found to overlap each other, and thus the gradual pressure of scales below, which are increasing in size, is the cause of the throwing off of these cuticular lamellæ. After these have been detached, their place is occupied by newly-formed scales.

Mr. Nasmyth remarks on another form in which the external layer of cuticle is removed, viz. in a continuous layer, as by frogs and efts. As soon as this layer is removed, another lamina of scales is seen on the surface of the animal's skin. If after the death of a frog it be immersed in water, this thin external translucent layer generally separates; but upon prolonging the maceration, another lamina is found to be gradually separating from the cutis, which is dense, and sometimes measures a quarter of a line in thickness. Internally it will be found to be composed of very numerous cells, while externally the regular series of scales is evident. The tessellated lamina alluded to above evidently takes its origin from this layer of cuticle. Mr. Nasmyth thinks that no other conclusion can be arrived at than that cuticle and epithelium are organised tissues. It would appear that they are formed from a fluid secretion on the surface of the vascular corion. The various stages of development being, 1, the formation of nuclei and corpuscles; 2, that of cells; 3, the growth of the latter effected by vital imbibition; 4, their compression and gradual conversion into minute lamellæ or scales. In short, it appears a rational conclusion that the component parts of the cuticle and epithelium have within themselves a power of growth; and it remains for pathologists to determine what share the derangement of this function has in the production of cutaneous diseases. Another argument in favour of the organic nature of the epithelium is derived from the circumstance, that under certain modifications it presents various vital phenomena, among which may be mentioned the ciliary motions.

Mr. Nasmyth has made the structure and development of that portion of the epithelium which lines the cavity of the mouth a subject of particular examination. In the fœtal subject, previous to the extrusion of the teeth, it forms on the alveolar arch a dense projecting layer, distinguishable from the surrounding membrane by its whiteness, and by the existence on its surface of ridges and sulci, having a waving course and a variable direction. The alveolar epithelium is thicker in proportion to the youth of the subject examined. It is most prominent where it corresponds with the molar teeth: its internal surface is concave, receiving the projecting mucous membrane. This portion presents various objects for investigation.

Firstly, as regards its composition:—It is made up of a mass of scales, lying one on the surface of the other. This disposition shows that the terms "dental cartilage," or the "cartilage of the gum," which have hitherto been applied to this structure, give an erroneous idea of its true nature, for cartilage always presents the corpuscle discovered and described by Purkinje. As in other portions of the epithelium, the external scales here are the larger, and this holds good generally, until we come to the surface of the vascular mucous membrane, which presents simple cells with their corpuscles.

In the interior of this alveolar epithelium, where it corresponds to the molar teeth, small vesicles may be frequently observed, varying in size from one quarter to one-eighth of a line in diameter. They appear to the naked eye to be transparent; under the microscope their parietes are found to consist of attenuated scales, and their cavity to contain a fluid abounding in minute granules and cells.* The internal surface of the epithelium covering the alveolar arch frequently presents concavities or indentations which are from a line and a half to three or four lines in circumference: they correspond to projections from the mucous membrane formed by a larger species of vesicle. The latter is deeply implanted in the vascular mucous membrane. The parietes of these vesicles are composed of a very delicate membrane; they contain a transparent fluid which coagulates on the application of heat, or acid, or on immersion in spirit, and in this fluid float numerous globules and scales similar to those of the epithelium generally. The internal or attached surface of the alveolar epithelium also presents numerous fringed processes measuring from one line to one and a half lines in length, and half a line in breadth, which sink into the substance of the subjacent mucous membrane. Under the microscope

---

* The vesicles here alluded to are most probably those which Serres describes as glands for the secretion of tartar: they are very numerous even after the extrusion of the incisor teeth of the calf, and are seen with great facility internally.

these fringes are found to be composed of elongated scales connected together, forming masses which divide and subdivide until they attain such an extreme tenuity that the most minute terminations consist but of two scales in marginal apposition. If the epithelium be carefully separated from the surface of the mucous membrane corresponding to the unextruded molar teeth, and placed in water or in diluted spirit of wine for some little time, its internal or attached surface presents these fringes much enlarged and forming a mass more considerable in size than the dense epithelium itself.

The epithelium covering the mucous membrane of the palate presents transverse rugæ, corresponding to those of the mucous membrane. If these palatal rugæ of the epithelium of the calf be carefully examined from the internal surface with a magnifying power of one inch focal distance, each will be found to consist, or to be composed of, numerous depressions or cul de sacs which receive prolongations or pointed processes of the subjacent mucous membrane.

They are of extreme tenuity, and, when viewed by the aid of high magnifying powers, are observed to consist of distinct scales.

Mr. Nasmyth is of opinion that mucus and epithelium are not, as has been supposed, identical.

Such is the substance of the three memoirs before us. Our readers are put in possession of the principal facts contained in them. They evince the laboriousness of Mr. Nasmyth's observations.

We can but glance at Mr. Owen's work, its partial publication and present incompleteness, precluding our giving any account of it.

We may state that, independently of an unfinished introduction, it comprises a history of the dental system of fishes, which is brought to a termination, and one of that of reptiles, which is merely commenced.

Of the execution of his task, we fancy that it is needless to speak. Mr. Owen's position, his opportunities, his labours, his zeal, and his acquirements, all offer a substantial and sufficient guarantee for first-rate performance. And a first-rate performance it is. When more is before the public we shall return to it, and notice it at ample length.

But we cannot quit the subject without recommending our readers to make themselves possessors of the work. The scientific and the curious will be fully repaid, and the library of every one of philosophical taste, and that of every public body, should possess it.

